# Assessment of flow pattern of right ventricle outflow and pulmonary arteries in surgically corrected tetralogy of Fallot patients by four-dimensional cardiac magnetic resonance flow

**DOI:** 10.1186/s43044-020-00092-y

**Published:** 2020-09-07

**Authors:** Mahmoud Shaaban, Mai Salama, Ayman Alsaied, Raghda Elsheikh, Magdy Elmasry

**Affiliations:** Cardiology Department, Faculty of Medicine, Tanta University, Tanta, Egypt

**Keywords:** 4D-CMR flow, 2D-PC flow, TOF, Pulmonary regurgitation

## Abstract

**Background:**

The most common post-surgical complication of tetralogy of Fallot (TOF) is pulmonary regurgitation (PR) which can lead to right ventricle (RV) dysfunction/failure. Cardiac magnetic resonance (CMR) is the imaging modality of choice to follow-up a repaired TOF. However, the conventional two-dimensional phase-contrast (2D-PC) flow usually underestimates PR as well as the pulmonary peak systolic velocity (PSV). Recently, four-dimensional (4D) CMR flow is introduced for more accurate quantitative flow assessment. This work aimed to compare between 4D-CMR and 2D-PC flow across the main (MPA), right (RPA), and left (LPA) pulmonary arteries (PAs) in surgically corrected TOF patients.

**Results:**

This study was conducted on 20 repaired TOF patients (range 3–9 years, 50% males). All patients had CMR exam on 1.5T scanner. 4D-CMR and 2D-PC flows were obtained at the proximal segments of the MPA, RPA, and LPA. The stroke volume index (SVI), regurgitation fraction (RF), and PSV measured by 4D-CMR were compared to 2D-PC flow. The SVI across the PAs was nearly similar between both methods (*P* = 0.179 for MPA, 0.218 for RPA, and 0.091 for LPA). However, the RF was significantly higher by 4D-CMR in comparison to 2D-PC flow (*P* = 0.027 for MPA, 0.039 for RPA, and 0.046 for LPA). The PSV as well was significantly higher by 4D-CMR flow (*P* = 0.003 for MPA, < 0.001 for RPA, and 0.002 for LPA). The Bland-Altman plots showed a good agreement between 4D-CMR and 2D-PC flow for the SVI, RF, and PSV across the pulmonary arteries.

**Conclusion:**

A good agreement existed between the two studied methods regarding pulmonary flow measurements. Because of its major advantage of performing a comprehensive flow assessment in a shorter time, 4D-CMR flow plays an important role in the assessment of patients with complex CHD especially in the pediatric group.

## Background

Tetralogy of Fallot (TOF) is one of the most common congenital heart disorder (CHD) and is the most common cyanotic CHD. Corrective surgery for TOF is usually performed in the first year of life [1]. It classically consists of ventricular septal defect (VSD) closure and reconstruction of the right ventricle outflow tract (RVOT) [2]. RVOT reconstruction can be performed by trans-annular patch, RV to pulmonary artery (PA) conduit, or a valve-sparing technique. The most common post-surgical complication is pulmonary regurgitation (PR) which can lead to secondary RV volume overload [3, 4].

The growing increment in endurance rate and the requirement for continuous clinical follow up are presently driving the need for improved cardiovascular imaging [1]. Cardiovascular magnetic resonance (CMR) is superior to echocardiography in the assessment of RV volume, quantification of PR, and monitoring the progression of PR and RV dilatation [5, 6]. Additionally, the new time-resolved three-dimensional (3D) phase contrast CMR (the also called four-dimensional (4D) CMR flow) has been proposed for more accurate quantification of cardiovascular blood flow and for obtaining more detailed information about vascular hemodynamics [7]. Accordingly, this method may be a promising method to precisely assess altered blood flow in patients with CHD with complex anatomy and hemodynamics [8–10].

4D-CMR flow is particularly important given that characterization of intra-cardiac flow is much more challenging than flow assessment in vessels due to the 3D geometry of the cavity and changing dynamics during the heart cycle [11]. In particular, the RV has a more complex shape than the left ventricle (LV), which can complicate flow assessment.

The aim of this study was to assess the flow across the main pulmonary artery (MPA), as well as the right (RPA) and left (LPA) pulmonary arteries in surgically corrected TOF patients by 4D-CMR flow and we compared the obtained results with the conventional two-dimensional phase contrast (2D-PC) flow.

## Methods

This study was conducted on 20 surgically repaired TOF patients who were presented for follow up at our institution in 2019. Eight patients were repaired with RVOT patch, 8 patients with trans-annular patch, and 4 patients with RV-PA conduit. CMR was performed on all included patients. A part of the 20 included patients, 8 patients were excluded from the study; 3 patients had atrial fibrillation affecting accurate flow measurements and the other 5 patients had poor CMR image quality. An informed written consent was obtained from care givers of all participants, and the study protocol was approved by the institutional ethics committee.

### Cardiac magnetic resonance imaging

CMR examinations were performed on 1.5T scanner (Siemens Magnetom Aera, Siemens Medical Systems, Erlangen, Germany) for all patients. The CMR exam included assessment of bi-ventricular volumes and systolic function, 2D-PC, and 4D-CMR flow across the MPA, RPA, and LPA.

### Bi-ventricular volumes and function

Steady-state free precession (SSFP) Cine images were typically used to measure ventricular volumes and function. Images were acquired in three long axes planes; Cine 4-Chamber, 2-Chamber, and 3-Chamber by retrospective electrocardiogram (ECG) gating throughout the whole cardiac cycle to assess wall motion abnormalities and ventricular functions. Continuous stacks of SSFP Cine images were acquired in axial plane covering the entire ventricles to quantify ventricular end-diastolic (ED) and end-systolic (ES) volumes, stroke volumes, and ejection fractions.

### 2D phase contrast flow

**Fig. 1:**
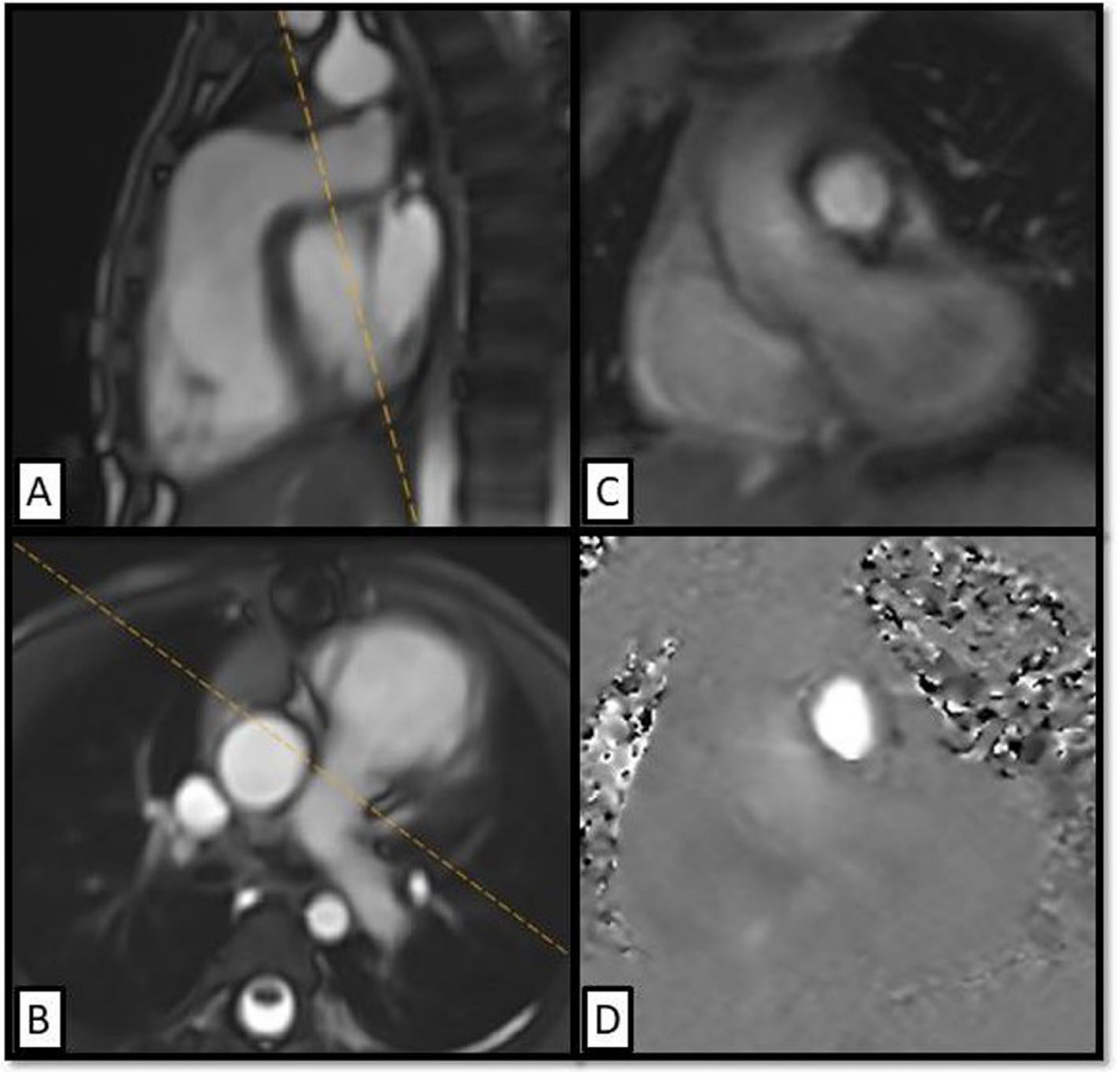
Planning of 2D-PC flow across the MPA. (**a**, **b**) Two orthogonal planes taken on sagittal (**a**) and coronal (**b**) SSFP Cine images of the MPA. **c** The resultant anatomy magnitude cross section image of the MPA and **d** is the PC image showing the forward systolic flow across the MPA (white color). PC: phase contrast, CMR: cardiac magnetic resonance, MPA: main pulmonary artery, SSFP: steady-state free precession

ECG triggered 2D-PC sequences were used for flow measurements and quantification of pulmonary regurgitation. The imaging planes were set perpendicular to flow in the main pulmonary artery as well as right and left pulmonary branches (Fig. 1). Flow data and the PR across the pulmonary vessels as well as the PSV across the MPA were measured using dedicated post processing software (Phillips intelliSpace Portal version 8.0).

### Four-dimensional CMR flow

#### 4D-CMR flow data acquisition

**Fig. 2:**
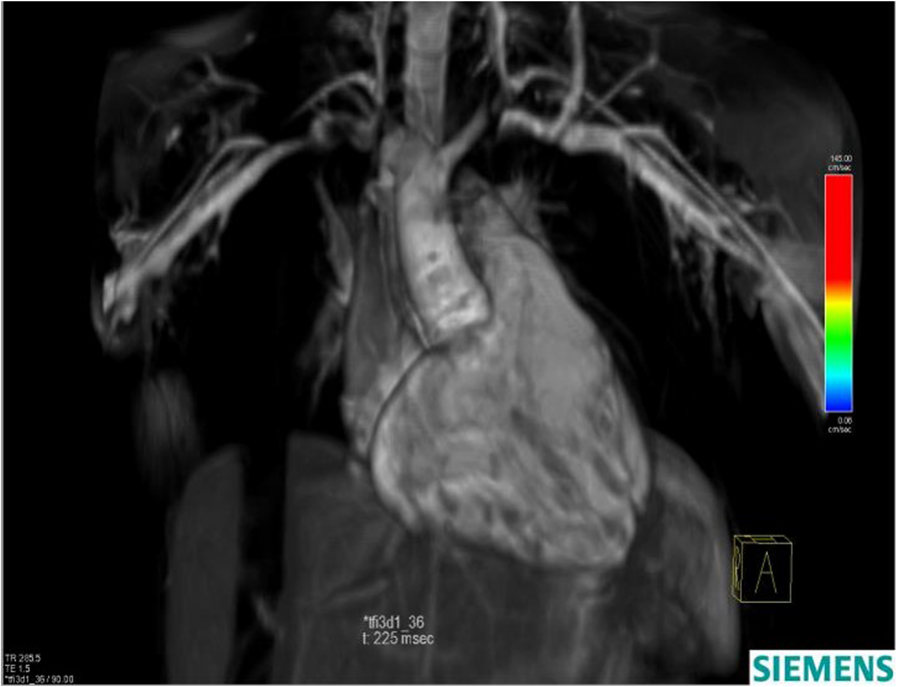
3D data volume acquisition in a surgically repaired TOF patient with RVOT patch. TOF: tetralogy of Fallot, RVOT: right ventricle outflow tract

4D-CMR flow acquisitions were synchronized to the heart rate using prospective ECG-gating and adaptive diaphragm navigator gating. The CMR sequence consisted of a k-space segmented rf-spoiled gradient echo sequence with interleaved three-directional velocity encoding. 3D data volume was individually adapted to include the entire RVOT, MPA, RPA, and LPA (Fig. 2).

#### 4D-CMR flow analysis

Reconstruction of magnitude images for anatomy identification and flow data images was performed by a dedicated 4D-CMR post-processing software (Siemens 4D flow V 2.4).

#### Qualitative flow analysis

**Fig. 3:**
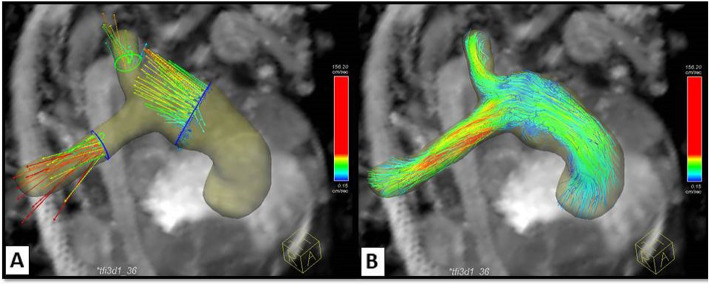
4D-CMR flow of the MPA, RPA, and LPA of a surgically repaired TOF patient with RV-PA conduit. **a** Vector field maps showing the velocity and direction of blood flow across pulmonary arteries during systole (arrows). This type of visualization provides a quick overview of velocity fields. **b** Streamlines color map showing also the forward flow across pulmonary arteries during systole. This type of visualization is useful to visualize 3D velocity fields at discrete time points. A high-systolic velocity across the RPA is noted by the two methods (red color map). CMR: cardiac magnetic resonance, MPA: main pulmonary artery, RPA: right pulmonary artery, LPA: left pulmonary artery, TOF: tetralogy of Fallot

Qualitative visualization of flow pattern across the RVOT and the PAs was done. Traces along the measured velocities were color-coded ranging from blue to red color according to the local velocity in the different vessels of interest with the lowest velocity given the blue color and the highest velocity given the red color. Assessment of the vortex formation and angulation of the flow across the cardiac cycle was performed. Multiple approaches were used to visualize flow, including color-coding mapping, vector fields, and streamlines (Fig. 3).

#### Quantitative flow analysis

**Fig. 4:**
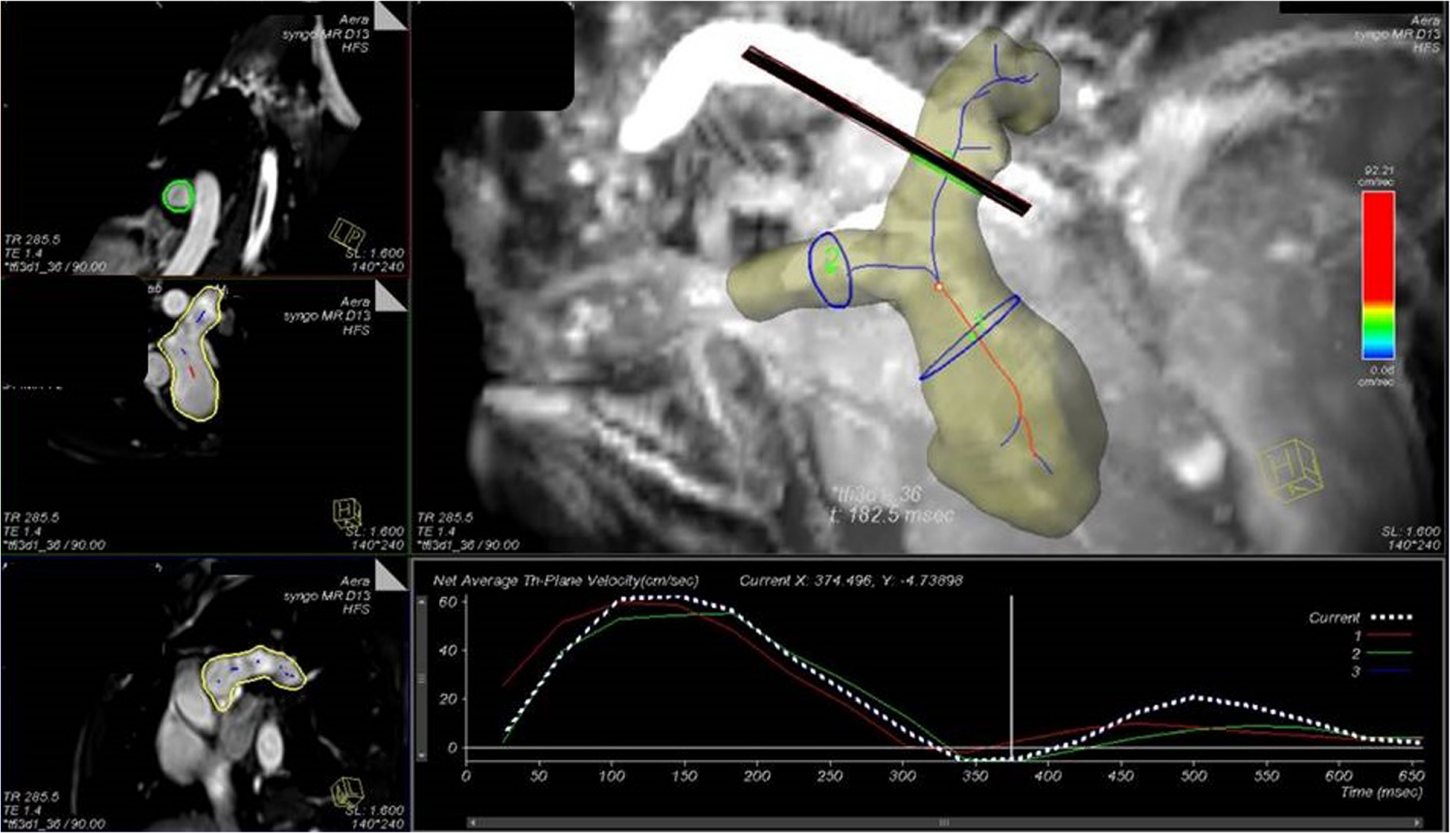
4D-CMR flow of the MPA, RPA, and LPA in a surgically repaired TOF patient with RVOT patch showing the offline planning of areas of interest at the proximal segments of these vessels to acquire all flow data and flow-time curves showed. CMR: cardiac magnetic resonance, MPA: main pulmonary artery, RPA: right pulmonary artery, LPA: left pulmonary artery

Flow-time curves, PR, and PSV across the pulmonary arteries were analyzed through manually positioned planes in the MPA just above the pulmonary valve, and in the proximal segments of the RPA and LPA (Fig. 4). Stroke volume (SV), RF, and PSV measured by 4D-CMR flow were compared to 2D-PC measurements.

## Statistical analysis

Statistical analysis was performed using IBM SPSS statistics 19.0.0 (SPSS Inc., Chicago, IL, USA). Nominal and continuous data were expressed as frequencies and mean ± standard deviation as appropriate. Non-parametric statistical hypothesis test (the Wilcoxon signed-rank test) was used to compare results obtained by 4D-CMR and 2D-PC flow. Bland-Altman plots were used to correlate between flow data obtained by 4D-CMR flow and 2D-PC flow.

## Results

Our study included 20 surgically repaired TOF patients (median age 4 years, range 3–9 years, 50% males, mean body surface area was 0.686 ± 0.143 m^2^). Eight patients (40%) had surgical repair with RVOT patch, 8 patients (40%) with trans-annular patch, and 4 patients (20%) were repaired with RV-PA conduit. The mean follow-up period by CMR after surgical correction was 2.4 ± 1.3 years. The RV EF was 56.3 ± 8.6%, RV EDVI 104 ± 23.5 ml/m^2^, RV ESVI 45 ± 15.9 ml/m^2^, and RV SVI 58.5 ± 15.7 ml/m^2^.

The SVI across the MPA, RPA, and LPA were nearly similar between 4D-CMR and 2D-PC flows with no statistically significant differences (44.87 ± 35.71 vs. 48.11 ± 41.79 ml/m^2^, 27.84 ± 17.11 vs. 30.46 ± 20.10 ml/m^2^, and 21.13 ± 17.11 vs. 22.92 ± 20.26 ml/m^2^, *P* = 0.179, 0.218, and 0.091 respectively) (Table 1).

**Table 1:** Comparison between the 4D-CMR and 2D-PC flows across the pulmonary arteries regarding the stroke volume index, regurgitation fraction, and peak systolic velocity

	4D-CMR flow	2D-PC flow	Significance paired *t* test
***MPA***			
SVI (ml/m^2^)	44.87 ± 35.71	48.11 ± 41.79	*P* = 0.179
RF (%)	24.25 ± 22.27	20.53 ± 18.98	***P*** = 0.027*
PSV (cm/s)	158.15 ± 28.78	148.34 ± 24.65	***P*** = 0.003*
***RPA***			
SVI (ml/m^2^)	27.84 ± 17.11	30.46 ± 20.10	*P* = 0.218
RF (%)	18.75 ± 19.69	14.29 ± 16.57	***P*** = 0.039*
PSV (cm/s)	160.09 ± 46.33	144.50 ± 46.98	***P*** < 0.001*
***LPA***			
SVI (ml/m^2^)	21.13 ± 17.11	22.92 ± 20.26	*P* = 0.091
RF (%)	26.36 ± 22.17	21.51 ± 18.95	***P*** = 0.046*
PSV (cm/s)	154.30 ± 40.62	136.36 ± 39.79	***P*** = 0.002*

*P P* value for Wilcoxon signed ranks test for comparing between 4D-CMR and 2D-PC flows

*Statistically significant at *P* ≤ 0.05

Regarding the RF across the MPA, RPA, and LPA, it was significantly higher by 4D-CMR compared to 2D-PC flow with statistically significant differences (24.25 ± 22.27 vs. 20.53 ± 18.98%, 18.75 ± 19.69 vs. 14.29 ± 16.57%, and 26.36 ± 22.17 vs. 21.51 ± 18.95 %, *P* = 0.027, 0.039, and 0.046, respectively) (Table 1).

Regarding the PSV across the MPA, RPA, and LPA, it was significantly higher by 4D-CMR compared to 2D-PC flow with statistically significant differences (158.15 ± 28.78 vs. 148.34 ± 24.65 cm/s, 160.09 ± 46.33 vs. 144.50 ± 46.98 cm/s, and 154.30 ± 40.62 vs. 136.36 ± 39.79 cm/s, *P* = 0.003, < 0.001, and 0.002, respectively) (Table 1).

**Fig. 5:**
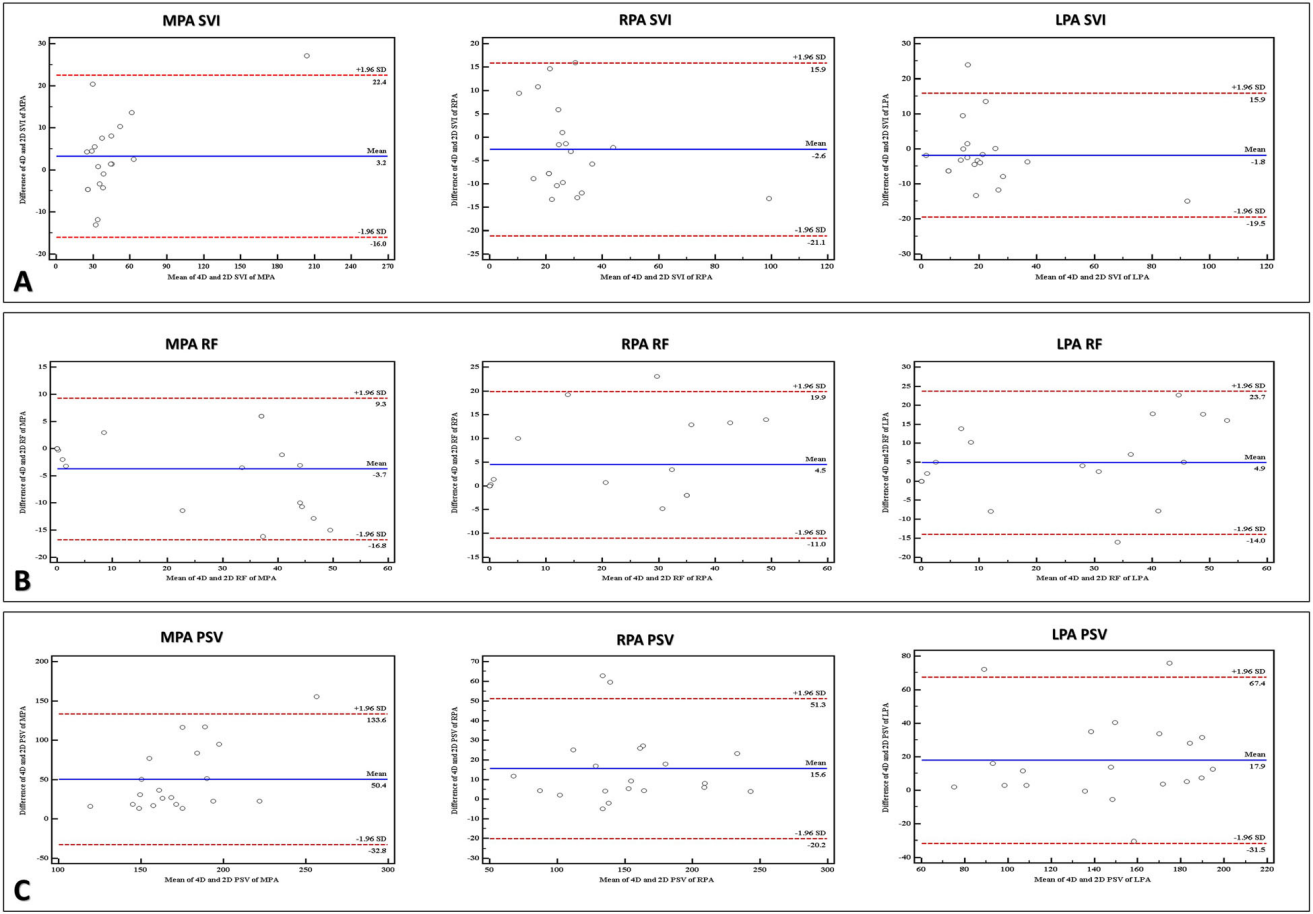
Bland-Altman plots comparing between 4D-CMR and 2D-PC flow across the pulmonary arteries. The middle line represents the mean difference while the upper and lower lines represent the ± 1.96 standard deviation limit. The plots show the lines of equality between and 95% limits of agreement between the two studied methods for the SVI (**a**), RF (**b**), and PSV (**c**) across the pulmonary arteries. SVI: stroke volume, RF: regurgitation fraction, PSV: peak systolic velocity, MPA: main pulmonary artery, RPA: right pulmonary artery, LPA: left pulmonary artery

Bland-Altman plots were used to correlate between the flow data obtained by 4D-CMR and 2D-PC flows and showed a good agreement between the two studied methods regarding the SVI, RF, and PSV across the MPA, RPA, and LPA as indicated by the data point distribution. (Fig. 5)

## Discussion

This study was conducted to assess the flow pattern across the pulmonary arteries in surgically corrected TOF patients by 4D-CMR flow and we compared it with the conventional 2D-PC flow.

The feasibility of 4D-CMR flow for the comprehensive assessment of flow patterns in pulmonary arteries and pulmonary hemodynamics was successfully demonstrated in this group of patients with surgically repaired TOF.

In our study, there was no significant difference in the SVI across the pulmonary arteries between 4D-CMR and 2D-PC flow while the RF was significantly higher by 4D-CMR flow in comparison to 2D-PC flow. However, there was a strong positive correlation between both methods regarding the SVI and RF across the pulmonary arteries.

Similar to our study, Jacobs et al. found a good agreement between 4D-CMR and 2D-CMR flows regarding the RF in pulmonary arteries [12]. Also, Raluca et al. [13] demonstrated a good correlation between both methods regarding flow volumes.

On the other side, Isorni et al. [14] found that 4D-CMR flow underestimated the pulmonary RF in comparison to 2D-PC flow in a study performed on 18 surgically repaired TOF patients. However, there was a good agreement between both methods.

Regarding the PSV across the pulmonary arteries, it was significantly higher by 4D-CMR flow in comparison to 2D-PC flow, yet with a strong positive correlation between both methods. Similarly, in a previous study performed by Nordmeyer et al. [15] which was conducted on 18 patients with semilunar valves stenosis, they reported higher PSV with 4D-CMR flow compared to 2D-PC flow when they were measured at the level with the highest velocity within the vessel and was more comparable to echocardiography [15].

Moreover, in another study performed by Gabbour et al. [16], they reported that 2D-PC flow significantly underestimated the PSV across MPA compared to echocardiography, while 4D-CMR was highly comparable to results obtained by echocardiography [16].

In the contrary, Raluca et al. [13] found that 4D-CMR flow underestimated the PSV when they performed the measurements at the same level as when using 2D-PC flow and they attributed this to the lower temporal resolution of 4D-CMR flow [13].

Finally, the major advantage of 4D-CMR flow which makes it superior to the conventional 2D-PC flow is that it acquires the entire volume in a single free-breathing acquisition. Additionally, flow can be measured anywhere and in any direction within the vessel of interest by prescribing infinite number of planes during offline analysis and the acquired data can be visualized for anatomy, flow velocity, volumes, and direction. In the contrary, 2D-PC flow requires separate acquisition of each vessel of interest with the probability of repeating it many times to enhance the plane in such complex CHD, further prolonging the scan time. In our study, the entire volume acquisition of 4D-CMR flow took on average 10 min while 2D-PC flow acquisition took much longer time for acquiring and optimizing the planes of interest within MPA, RPA, and LPA. Saving scan time is of extreme importance in the assessment of patients with complex CHD especially in the pediatric group.

## Study limitations

This study is limited by the relatively small number of patients. Larger patient cohorts and further follow-up examinations are essential to learn more about altered pulmonary hemodynamics following TOF repair.

Another limitation of this study is the absence of a gold standard for pulmonary flow analysis to be correlated with the two studied methods.

## Conclusion

There was a good agreement between 4D-CMR and 2D-PC flow regarding pulmonary flow measurements. Because of its major advantage of performing a comprehensive flow assessment in a shorter time, 4D-CMR flow plays an important role in the assessment of patients with complex CHD especially in the pediatric group. Further studies on larger patient cohort are needed to determine its added value in the assessment of right-sided hemodynamics.

## Data Availability

The datasets used during the current study are available from the corresponding author on reasonable request.

## Abbreviations

2D: Two dimensional
3D: Three dimensional
4D: Four dimensional
CHD: Congenital heart disease
CMR: Cardiovascular magnetic resonance
ED: End-diastolic
ES: End-systolic
LPA: Left pulmonary artery
LV: Left ventricle
MPA: Main pulmonary artery
PA: Pulmonary artery
PC: Phase contrast
PSV: Peak systolic velocity
RF: Regurgitation fraction
RPA: Right pulmonary artery
PR: Pulmonary regurgitation
RV: Right ventricle
RVOT: Right ventricle outflow tract
SSFP: Steady state free precession
SV: Stroke volume
TOF: Tetralogy of Fallot
VSD: Ventricular septal defect
